# Cowpea (*Vigna unguiculata (L.) Walp*) for food security: an evaluation of end-user traits of improved varieties in Swaziland

**DOI:** 10.1038/s41598-019-52360-w

**Published:** 2019-11-05

**Authors:** Therese Mwatitha Gondwe, Emmanuel Oladeji Alamu, Phumzile Mdziniso, Busie Maziya-Dixon

**Affiliations:** 1 Food and Nutrition Sciences Laboratory, International Institute of Tropical Agriculture (IITA),PMB 5320, Oyo Road, Ibadan, Oyo State, Nigeria; 2 Department of Agricultural Research and Specialists Services, Malkerns Agriculture Research Station, Malkerns, Swaziland

**Keywords:** Biophysical chemistry, Biophysical chemistry

## Abstract

Improved varieties have agronomic advantages over local varieties,but not much attention has been given to understand the nutritional content of the improved cowpea varieties released in Swaziland. This study investigated the physical and nutritional properties of improved cowpea varieties released in Swaziland. Five improved varieties (IT-04K-321-2, IT-97K-390-2, IT-18, IT-16, and IT-99K-494-6) and one local variety (Mtilane, as check) were analyzed for physical and chemical properties. The results showed that there were variations in seed weight as the values ranged between 12.5 and 18.5 g per 100 g seed weight. The protein content ranged from 25.38% to 27.56% with no significant difference (P < 0.05) between the improved varieties  and the local variety, ash content ranged between 3.47 and 6.84%, crude fiber was between 5.81 and 15.08%, and carbohydrate ranged from 45.64 to 57.12%.  Contents of calcium ranged from 9 to 36 mg/100 g and of iron from  2.0 to 2.4 mg/kg, with significant differences (P  < 0.05)  between the varieties with highest and the lowest values. Zinc content ranged from 7 mg/kg to 8 mg/kg, with no significant difference (P >0.05) among the varieties. The improved varieties have high seed weight, which is an essential factor that farmers consider when choosing a variety to adopt. In terms of addressing nutritional security, the crop is suitable for addressing protein-energy malnutrition and formulating blends for baby foods in Swaziland.

## Introduction

Cowpea (*Vigna unguiculata*) is often referred to as the poor man’s meat as it is a significant source of protein, minerals, and vitamins^1^ for the rural poor who have limited access to protein from animal sources such as meat and fish^2^. The cowpea plant is a drought-tolerant food crop, well adapted to a diverse range of climate and soil types, and widely cultivated throughout the tropics and subtropics of Africa, Latin America, and Southeast Asia, as well as in the United States^3^. In Africa, cowpea is mainly cultivated in West and Central Africa, with an annual production of 3 million tons^4^. In Swaziland, cowpea plays a significant role in the diets of rural and urban communities, and is mostly consumed in combination with starchy foods such as pap or maize grits in the form of samp (maize gruel) and *siphuphe* (mashed cowpeas). In other countries, the grain has been used to fortify cereal-based weaning foods, in which it forms complementary amino acid profiles and improved protein quality^6^. The crop is, therefore, gaining industrial importance in food formulation^5^ from its nutritional and functional benefits. Also, McWatters *et al*.^7^ prepared biscuits with good sensory quality from cowpea composite flour. Consumption of legumes has been associated in many clinical studies^8–10^ with a reduction in cholesterol and the risks associated with coronary heart diseases. Whether improvements in the crop production performance of cowpea have also led to changes in the nutritional and physical characteristics of the grainhas, however, not been documented.

In Swaziland, cowpea is mainly grown for its grain because of its ability to tolerate dry spells or drought. Like most of the Southern African countries, Swaziland has experienced the effects of climate change manifested in the form of drought and the short duration of rainfall. The Government is promoting the cultivation of improved cowpeas because grain yields are higher than from local varieties. Cowpea breeding research prioritizes improvement in the yields as the most important trait. Less attention has been given to the relationship between high-yielding varieties and grain quality. The Swaziland national program is intensifying research on evaluating high yielding varieties and disseminating the adaptability of early maturing cowpea to improve production and promote marketing. Some studies have been done to analyze the relationship between seed type and the effect on physical and chemical attributes^11,12^. With this background the present study was commissioned to determine and compare the physical and chemical properties of the improved cowpea lines against  the local variety. Knowledge generated from this study would be used by researchers, processors, dieticians, and policymakers in planning (for example) hospital and school-feeding programs where there would need to match varieties to specific purposes for various needs^12^ based on their chemical characteristics.

## Materials and Methods

The cowpea materials used for this study were planted and harvested during the 2014/2015 cropping season at Malkerns Research Station in Swaziland. Soil samples were collected at a depth of 0–20 cm before planting. Soil samples were air-dried and stored under appropriate conditions (in the refrigerator) for nutrient analysis. Each plot was 2.5 m wide and 5 m long with  five0 rows of 5 m and a total area of 7.5 m^2^. The alley between plants was 1 m, and 2 m  between replications. The Randomized Complete Block Design with three replications, was used to install the trial. Treatments included five improved varieties (IT-04K-321-2, IT-97K-390-2, IT-18, IT-16 and IT-99K-494-6) and one local variety (Mtilane, as check). Two seeds were planted per station at 10 cm between stations. The planting depth was 3 cm. Weeding was done twice during the planting season to ensure weed-free conditions. Grain yield was determined by weighing seed/plot and yield/ha was calculated, based on the area harvested. The cowpea samples were processed into flour using the adapted method of Alamu *et al*.^13^. Clean and sorted cowpea grains of a representative sample of each variety were carefully selected, milled (sieve size, 0.5 mm), packed in a well-labeled polyethylene whirl- pack and stored at 4 °C prior to analysis. All three replications from the field were sampled for laboratory analysis.

### Determination of physical properties

 The cowpea varieties used for the assessment (Table 1) were five IITA lines: IT-04K-321-2, IT-97K-390-2, IT-18, IT-16, and IT-99K-494-6 that were released in Swaziland in 2015, plus Mitlane, another common variety found in Swaziland, as a local check. Physical properties were estimated for each of the six varieties using the following methods:

- Seed weight (g):100 seeds of each variety were randomly selected and weight (g) was recorded.
- Seed color: The seed color was determined visually and from breeder’s information that was provided during the application for release.

**Table 1:** Physical characteristics of newly released cowpea varieties with the local check.

Cultivar	Seed coat colour	100 seed weight (g)	Grain yield (t/ha)
IT-04K-321-2	White	27.00a	1.91a
IT-97K-390-2	Reddish -brown	14.50bc	1.12b
IT – 18	Brown	12.50c	0.92b
IT – 16	Reddish–brown	15.50bc	0.81b
MTILANE (local check)	Reddish	14.50bc	0.80b
IT-99K-494-6	Brown	18.50b	0.79b
MEAN		17.08	1.06
LSD		4.92	0.70
CV (%)		19.11	31.54
Max		27.5	1.12
Min		12.5	0.79

Means with the same letters are not significantly different (p = 0.05).

### Determination of proximate composition

Moisture, ash, fat, crude fibre (CF) and crude protein (CP) contents were determined using the methods described by Alamu *et al*.^13^ and the Association of Official and Analytical Chemist (AOAC)^14^. The  total carbohydrate content was calculated by difference using the method described by Merrill and Watt^15^. The loss in weight after drying for 24 hours in Fisher Scientific Isotemp® Oven model 655 F was recorded as moisture content. The ash content was estimated after moisture, and all organic constituents were burned off at 600 °C. Crude fat was extracted from the sample with hexane, and the solvent was evaporated off to get the fat. CP content was determined by the Kjeldahl method using Kjeltec™ Model 2300, as described in Foss Analytical AB manual^16^. A conversion factor of 6.25 was used to convert from total nitrogen to the percentage of CP.

### Determination of calcium, iron, and zinc

Iron (Fe) and zinc (Zn) contents were determined using the method described in AOAC^14^. Five grams (5 g) of each flour sample was gently heated over a Bunsen burner flame until most of the organic matter was destroyed. The remaining material was further exposed to high temperatures in a muffle furnace for several hours until white-grey ash was obtained and then cooled. About 20 ml of distilled water and 10 ml of dilute hydrochloric acid were added to the ash material. This mixture was boiled, filtered into a 250 ml volumetric flask, washed thoroughly with hot water, cooled, and made up to volume.  Contents of Fe and Zn in each sample were analyzed using Atomic Absorption Spectrophotometer (PYE Unicon, UK, and Model SP9).

### Statistical analysis

All laboratory analyses were done in duplicate; the mean ± standard deviation of the results from the experiment was calculated using the statistical analysis system (SAS) software package version 9.3. Duncan’s multiple range test was used to separate the differences in the mean scores at a significance level of P = 0.05.

## Results and Discussions

### Physical characteristics and yield performance of the varieties

The physical characteristics of the six cowpea varieties are given in Table 1.

#### Seed coat color

The analyzed varieties had the following colors:white, reddish-brown, brown, and reddish, as shown in Table 1. Seed color in cowpea is an essential feature because it directly influences the marketability of the grain^17^.

#### Seed weight and size

The value of 100 seed weight was significantly higher for IT-04K-321-2 (27) than the other varieties. The results are similar to the findings of Kabambe *et al*.^18^, who found that IITA-improved varieties were superior in terms of seed weight. This is a vital marketing trait as the   heavier the seeds, the better the price they command. In processing, seed weight is also an essential information for designing the appropriate processing machines that could handle a maximum weight of seeds. Seed size in cowpea is crucial because it directly indicates the productivity of the variety and, together with color standards, determines grain quality for commercialization^17^. For example, in northeast Brazil, consumers prefer medium to large grain sizes (15 g to 25 g). In most of the cowpea lines, the seed size varies, some varieties weigh less than 10 g per 100 seeds and some weigh approximately 30g^19^. The size of the grains indicates the quality of seeds because vegetative growth is usually affected by the initial quality of the seeds that were planted. Good quality seeds contribute15–20% to increases in yield ^20^. Also, El-Sawah^21^ and Nosser & Behnan^22^ found that dense seeds positively affect the chemical composition of plants such as N, P, and K and the quality of protein and total soluble carbohydrates.

#### Grain yield

Grain yield was determined by weighing harvested seeds/ plot and calculated yield/ hectare was based on the area harvested. An improved variety (IT-04K-321-2) differed significantly at P = 0.5 from the other varieties in terms of seed weight and performance in the field (yield/ hectare) as shown in Table 1.

### Proximate composition of cowpea varieties

Results from proximate analysis (Table 2) indicate that the moisture content (MC) of the tested varieties ranged from a minimum of 10.68% to a maximum of 10.90%, with only two of the improved varieties (IT-04K-321-2 and IT-97K-390-2) significantly (P < 0.05) different from the local check (Mtilane). The result of this study agrees with the report of Singh^11^. The low moisture content of these varieties is an indication of good storage life.

**Table 2:** Proximate composition (%) of cowpea varieties.

Variety	Moisture content	Ash	Crude protein	Fat	Crude Fibre	Carbohydrate
IT-04K-321-2	10.90 ± 0.14^a^	3.05 ± 0.09^c^	27.13 ± 1.01^a^	1.26 ± 0.69^a^	15.08 ± 0.13^a^	45.64 ± 1.15^e^
IT-16	10.68 ± 0.10^a,b^	3.09 ± 0.02^c^	27.13 ± 1.01^a^	0.87 ± 0.19^a^	8.14 ± 0.03^e^	53.19 ± 1.26^b^
IT-18	10.70 ± 0.12^ab^	3.26 ± 0.01^b^	27.13 ± 1.01^a^	0.81 ± 0.10^a^	10.64 ± 0.08^d^	50.72 ± 1.10^c^
IT-97K-390-2	10.90 ± 0.08^a^	3.47 ± 0.02^a^	25.38 ± 1.01^a^	0.79 ± 0.30^a^	5.81 ± 0.03^f^	57.12 ± 0.93^a^
IT-99K-494-6	10.80 ± 0.24^ab^	2.88 ± 0.13^d^	26.25 ± 0.00^a^	1.16 ± 0.38^a^	13.88 ± 0.25^b^	47.91 ± 0.44^d^
Mtilane	10.60 ± 0.00^b^	2.97 ± 0.08^cd^	27.56 ± 1.68^a^	0.60 ± 0.12^a^	12.64 ± 0.32^c^	48.60 ± 1.72^d^
Min	10.68	2.97	25.38	0.60	5.81	45.64
Max	10.90	3.47	27.56	1.26	15.08	57.12
CV	1.15	6.84	3.00	27.07	32.14	8.16
Pr > F	*	***	ns	ns	***	**

Means with the same letters are not significantly different p = 0.05; ns = not significant.

The results from analysis showed that ash content among the varieties ranged from 2.97% to 3.47%, an indication that they are a good source of minerals. All the tested varieties except IT-99K-494-6 showed a higher ash content (above 3%) than the local check. IT-18 and IT-97K-390-2 differed significantly (P < 0.05) from the rest with higher values. However, these values are slightly lower than those reported by Ajeigbe *et al*.^12^.

 The CP ranged from 25.38% to 27.56%, and there was no significant difference between the improved varieties and the local variety in terms of protein content. This finding agrees with those reported by authors^12,23,24^, who found that the protein content of most of the IITA-improved varieties ranged between 20% and 27%. However, varieties IT-04K-321-2, IT-16, and IT-18 compared favorably with the local variety (Mtilane) with protein content above 27%. The high protein contents in these varieties could address high levels of protein deficiency such as kwashiorkor. For Swaziland, 31% of the children under-five are stunted, 5.9% are underweight, and 1% wasted^25^.

Results for CF indicate a range from 5.81% to 15.08%. The highest CF content was observed for variety IT-04K-321-2. There were highly significant differences (P < 0.05) among all improved varieties compared with the  local check. The CF values recorded in this study are higher than those reported by Osunbitan *et al*.^26^ and Ajeigbe *et al*.^12^. The higher CF content in these varieties could vary depending on the interaction between the genetic make-up and the growing environment^27^. These varieties could be excellent sources of dietary fiber and are very useful in adding bulk to food to relieve constipation as supported by Appiah *et al*.^3^. The fat content ranged from 0.60% to 1.26%. Highest fat content (1.26%) was observed in IT-04K-321-2, and the lowest fat content was found in the local variety Mtilane. However, there were no significant differences (P > 0.05) among all the varieties and between the improved and the local variety. The differences in the composition of cowpea could be attributed to soil type, cultural practices, environmental conditions, and genetic makeup^28^.

The carbohydrate content ranged from 45.64 to 57.12%, with IT-97K-390-2 having the highest carbohydrate content. The carbohydrate content is within the range reported by Ashogbon and Akintayo^29^ whose values are of about 50% and above, except for one improved variety (IT-04k-321-2) which showed a slightly lower value of 45.64%.

### Calcium, iron, and zinc contents of cowpea varieties

Results on calcium, iron, and zinc are shown in Table 3. The analysis revealed that all varieties, except IT 99-494-6, differed significantly from the local check, having higher levels of calcium than Mtilane. Calcium content ranged from 9.0 to 36.0 mg/100 g, with highest levels found in IT-18. All varieties, except IT-99-494-6, differed significantly from the local check. The differences in calcium content could be attributed to the genetic makeup of the varieties and the calcium content of the soil. Similar findings were reported in a study in Nigeria, where there were variations in the amount of calcium present in different cowpea varieties. It is noted in this study that the noted differences in calcium could be due to soil type and location where these cowpea varieties were grown because the soil’s mineral content influences the mineral uptake of the plants^26^. However, the varieties that were used in this study were planted in the same location, and so the differences in calcium content could be attributed to the genetic makeup of the varieties. In terms of the values, the calcium content of these varieties is relatively lower (9.0 –36.0 mg/100 g) than in IITA improved lines such as IT97K-499-8 (68.48 mg/100 g) and IT96D −773 (63.0 mg/100 g)^25^. Calcium also plays a vital role in muscle contraction, transmitting messages through the nerves, and in the release of hormones. If people are not getting enough calcium in their diet, the body takes calcium from the bones to ensure normal cell function, and this  may lead to weak bones.

**Table 3:** Calcium, iron, and zinc composition of cowpea varieties.

Variety	Calcium (mg/100 g)	Iron (mg/ kg)	Zinc(mg/kg)
IT-04K-321-2	23.0 ± 0.01^d^	2.1 ± 0.01^ab^	0.7 ± 0.01^a^
IT-16	30.0 ± 0.02^b^	2.4 ± 0.01^a^	0.7 ± 0.01^a^
IT-18	36.0 ± 0.02^a^	2.1 ± 0.01^ab^	0.8 ± 0.01^a^
IT-97K-390-2	9.0 ± 0.02^e^	2.4 ± 0.01^a^	0.7 ± 0.01^a^
IT-99K-494-6	28.0 ± 0.02^bc^	2.0 ± 0.01^b^	0.7 ± 0.01^a^
Mtilane	26.0 ± 0.02^c^	2.0 ± 0.02^b^	0.7 ± 0.00^a^
Min	9.0	2.0	0.7
Max	36.0	2.4	0.8
CV (%)	36.00	57.04	5.70
Pr > F	***	**	ns

 The presence of iron  and zinc in all the cowpea varieties is vital as these are micronutrients responsible for essential body functions, and a deficiency in these minerals can lead to severe medical conditions^30^. The iron content ranged from 2.0 to 2.4 mg/k g, with significant differences (P < 0.05) between the varieties with highest and the lowest values. The highest concentrations were found in IT-16 and IT-97K-390-2, and the lowest in IT-99K-494-6 and Mtilane. Iron is needed for the transfer of oxygen to body tissues and other organs^31^. Zinc content ranged from 7 mg/kg to 8 mg/kg, with no significant difference among all varieties. This range of values is similar to reports by Central Statistics Office (CSO) and UNICEF^25^ for a study that was done in Iringa location, Tanzania. Zinc plays an essential role in the body in terms of metabolism, and it prevents illnesses by supporting the immune system^31^. The results show no significant differences between the local and the improved varieties in terms of iron andzinc, indicating that the improved varieties are as nutritionally good as the local cowpea variety despite their differences in agronomic traits such as yield and disease tolerance. The findings are in agreement with Mamiro *et al*.^24^.

### The correlation coefficient between mineral and chemical properties of cowpea varieties

The correlation coefficients among different mineral and chemical properties of cowpea varieties are given in Table 4. There were significant differences in the following properties : moisture content and calcium had a negative correlation (r = − 0.49, ≥0.05); ash and calcium correlated negatively (r = − 0.47, P ≥ 0.05). There was a significant and robust positive correlation between ash and iron (r = 0.52, P ≥ 0.01), which shows that breeders could breed varieties with high ash and iron content as iron is an essential nutrient in the body. There was a negative but strong correlation between CF and iron (r = − 0.66, P ≥ 0.01); therefore, breeders could breed varieties that are high in iron and low in CF. Similarly, a robust but negative correlation between CF and ash (r = − 0.75, P ≥0.001); and a positive correlation between protein and calcium (r = 0.43, P ≥ 0.05), which indicates that protein content increases as calcium increases, and both are essential nutrients especially for children and the elderly. Therefore, breeders could breed varieties that are high in both protein and calcium, while food scientists could develop cowpea-based products that are rich in both nutrients.

**Table 4:** Correlation between mineral and chemical properties of cowpea varieties.

	Fat	Zinc	Calcium	Iron	MC	Ash	CF	Protein
**Pearson Correlation Coefficients, N = 24**								
Fat	1.00000							
Zinc	−0.13944	1.00000						
Calcium	0.05491	0.07202	1.00000					
Iron	0.05624	−0.02954	−0.34073	1.00000				
Moisture content	0.07955	−0.16991	−0.49165*	0.10292	1.00000			
Ash	−0.08988	0.10707	−0.47028*	0.52818**	0.23822	1.00000		
Crude fiber	0.32511	−0.09489	0.39263	−0.66170**	−0.01876	−0.75676***	1.00000	
Protein	−0.02441	−0.15253	0.43791*	−0.34210	−0.24143	−0.34158	0.32158	1.00000

## Conclusion

The results of this study have shown that the improved cowpea lines that were released in Swaziland have a high seed weight that is an important factor for  the  design of industrial grain processing machines for both small and medium scale cowpea processors. In terms of nutritional content, the studied varieties have high protein and carbohydrate contents, and would be suitable for addressing protein-energy malnutrition as well as in formulating blends for baby foods. All tested varieties were found to be low in fat and high in CF, which means that they would be suitablefor  formulating foods for people with diabetes.
